# The Effect of Autologous Protein Solution on the Inflammatory Cascade in Stimulated Equine Chondrocytes

**DOI:** 10.3389/fvets.2019.00064

**Published:** 2019-03-06

**Authors:** Renata L. Linardi, Michael E. Dodson, Kaitlyn L. Moss, William J. King, Kyla F. Ortved

**Affiliations:** ^1^Department of Clinical Studies, New Bolton Center, University of Pennsylvania, Kennett Square, PA, United States; ^2^Owl Manor Veterinary Inc., Warsaw, IN, United States

**Keywords:** autologous protein solution, osteoarthritis, equine, orthobiologics, joint disease, autologous conditioned serum, PTOA, chondrocyte

## Abstract

Cartilage injury occurs commonly in equine athletes, often precipitating posttraumatic osteoarthritis (PTOA). Orthobiologics such as autologous conditioned serum (ACS) and autologous protein solution (APS) may be useful in decreasing posttraumatic inflammation, thereby preventing PTOA. The objective of this study was to quantify cytokine concentrations in ACS and APS and evaluate the protective effects of ACS and APS on inflamed chondrocytes cultured *in vitro*. We hypothesized that the combination of platelet-derived growth factors (PDGF) and anti-inflammatory cytokines present in APS would be superior in decreasing the inflammatory and catabolic cascade in inflamed chondrocytes when compared to ACS in which platelets are excluded from the preparation. Chondrocytes were isolated from the cartilage of femoral trochlear ridges of 6 horses and cultured in 12-well transwell plates. Treatment groups included: (1) control, (2) APS (Pro-Stride; Owl Manor), and (3) ACS (IRAP II; Arthrex). Each group was unstimulated or stimulated with IL-1β and TNF-α for 48 h. The concentration of IL-1β, IL-6, TNF-α, MMP-3, MMP-13, and IL-10 was quantified using a fluorescent bead-based multiplex assay. IL-1Ra concentration was quantified using ELISA. APS and ACS both had significantly increased concentrations of IL-1Ra without a concurrent increase in IL-1β concentration. After 48 h of culture, media from chondrocytes treated with APS contained significantly increased concentrations of IL-1Ra and IL-10. APS-treated cultures had increased concentrations of IL-6. Overall, APS effectively concentrated IL-1Ra without an incubation period and media from APS-treated chondrocytes had increased concentrations of chondroprotective (IL-1Ra and IL-10) and modulatory (IL-6) cytokines, which may be beneficial in the treatment of inflammatory conditions such as PTOA.

## Introduction

Traumatic injury to articular cartilage occurs commonly in the equine athlete and, due to the poor intrinsic healing capabilities of cartilage, can lead to osteoarthritis (OA) (1, 2). Following injury, posttraumatic inflammation occurs within the joint characterized by increased activity of catabolic cytokines and decreased activity of anabolic cytokines (3). Uncontrolled inflammation leads to degradation of the extracellular matrix (ECM) and eventually, posttraumatic osteoarthritis (PTOA). PTOA is a progressive, debilitating disease that is the most prevalent musculoskeletal disease in horses and is responsible for substantial economic loss in the equine industry (4).

Interleukin-1β (IL-1β) produced by inflamed chondrocytes and synoviocytes, is a central cytokine driving inflammation and leads to upregulation of degradative enzymes, including matrix metalloproteinases (MMPs) and aggrecanases, that actively degrade proteoglycans, and type II collagen in the ECM. Traditional methods for controlling joint inflammation and pain include administration of intra-articular corticosteroids and hyaluronic acid; however, more recently autologous blood products have been investigated as they aim to decrease inflammation while stimulating healing. Additionally, the detrimental effects of long-term intra-articular corticosteroid administration can be avoided.

Platelet-rich plasma (PRP) and autologous conditioned serum (ACS) were two of the first orthobiologics to be marketed for the equine patient. PRP is prepared through centrifugation or filtration of the patient's blood to concentrate platelets. Its efficacy is based on degranulation of platelet α-granules leading to a release of a milieu of growth factors including platelet-derived growth factor (PDGF), transforming growth factor-β (TGF-β), fibroblast growth factor (FGF), vascular endothelial growth factor (VEGF), among others, that help modulate the healing response in damaged tissue (5). Autologous conditioned serum is similarly produced from whole blood; however, the main goal of ACS is to concentrate endogenous proteins, namely interleukin-1 receptor antagonist protein (IL-1Ra), produced by incubated white blood cells (6). Both PRP and ACS have been used to treat OA in horses and humans with some reported success (7–10).

Recently, a new patient-side product has been made available that aims to combine the benefits of ACS and PRP. Autologous protein solution (APS) concentrates platelets, growth factors, and anti-inflammatory cytokines through a two-step centrifugation process. Unlike ACS, production of APS does not require a 24-h incubation period, making it a more appealing product for equine veterinarians. The aim of this study was to quantify the concentration of several cytokines in APS and evaluate the effect of APS on the inflammatory cascade in chondrocytes cultured *in vitro*. We hypothesized that the combination of PDGF and anti-inflammatory cytokines present in APS would be superior in decreasing the inflammatory and catabolic cascade in inflamed chondrocytes when compared to ACS in which platelets are excluded from the preparation.

## Methods

### Blood Collection

Six young (2–8 years) adult horses free of systemic disease and being euthanized for reasons unrelated to the study were used. The study was performed in accordance with Institutional and NIH guidelines for the Care and Use of Laboratory Animals and the study was approved by the Institution Animal Care and Use Committee (IACUC) at the University of Pennsylvania. Prior to euthanasia, blood was sterilely collected from the jugular vein for preparation of ACS (IRAP-II™; Arthrex, Naples, FL), APS (Pro-Stride™; Owl Manor, Warsaw, IN), and serum. Both ACS and APS products were prepared according to manufacturer's instructions. Briefly, ACS was generated using the Arthrex IRAP II kit from venous blood (50 mL) obtained from a sterilely prepared jugular vein. Following collection, blood was transferred to an IRAP II® syringe containing borosilicate beads and incubated at 37°C for 24 h. The incubated syringe was then placed into a centrifuge and spun at 3,200 RPM (2,790 g) for 10 min. Autologous protein solution was generated using the Owl Manor Veterinary Pro-Stride kit from venous blood (55 mL) obtained from a sterilely prepared jugular vein into a syringe containing acid citrate dextrose (ACD) anticoagulant (5 mL). Following collection, the blood was transferred to the APS Separator and centrifuged at 2,000 RPM (1,744 g) for 15 min. Platelet-poor plasma was drawn off and then the cell solution was transferred to the APS Concentrator containing polyacrylamide beads, which was then centrifuged at 681 g for 2 min. For serum preparation, 8 mL of whole blood was collected into a plain blood tube without anticoagulant. Blood was left undisturbed for 60 min and then centrifuged at 2,000 g for 10 min. Serum was collected following centrifugation. All blood products including ACS, APS, and serum were stored for use at −80°C prior to use in the cell culture assay. An aliquot was stored for cytokine characterization.

### Cartilage Harvest

Following euthanasia, cartilage was harvested from the articular surface of the lateral and medial femoral trochlear ridges of horses and digested in 0.075% collagenase as previously described (11). Chondrocytes (100,000 cells/well) were cultured in 12-well transwell plates (Corning Inc., Corning, NY) in Ham's F12 medium (Gibco-Life Technology, Grand Island, NY) supplemented with 10% fetal bovine serum (FBS), 50 μg/mL ascorbic acid, 30 μg/mL α-ketoglutarate, 300 μg/mL L-glutamine, 100 units/mL penicillin/streptomycin, and 25 mM HEPES (Gibco-Life Technology, Grand Island, NY) and allowed to adhere for 96 h prior to any treatment.

### Chondrocyte Treatment

A total of six treatment groups were included: (1) Control (untreated media), (2) ACS, and (3) APS. Within each treatment group chondrocytes were either unstimulated or stimulated with IL-1β + TNF-α. Two hours prior to treatment, all medium was changed to serum-free medium (Opti-MEM; Gibco-Life Technology, Grand Island, NY) and ACS (0.5 mL) or APS (0.5 mL) was added to the top portion of each transwell insert. Opti-MEM (0.5 mL) was added to the transwell in the control wells. Following the above 2-h incubation, cells were stimulated with IL-1β (10 ng/mL) + TNF-α (1 ng/mL) for 48 h. All treatments were performed in duplicate.

### Cytokine Analysis

Medium was collected from each well at the end of 48 h. The concentration of MMP-3, MMP-13, TGF-β, IL-1β, TNF-α, IL-6, and IL-10 was quantified using a fluorescent bead-based multiplex assay (Luminex, Austin, TX). Antibodies against MMP-3, MMP-13, and TGF-β were anti-human, whereas all other were anti-equine. IL-1Ra concentration was quantified using an equine-specific sandwich ELISA (R&D Systems, Minneapolis, MN). For the IL-1Ra ELISA, the assay was performed according to the manufacturer's protocol with the following changes. Following collection of serum, ACS, or APS, 1.4 μL (500 × dilution) of protease inhibitor cocktail (AbCam, Cambridge, MA) and 70 μL (10 × dilution) of radioimmunoprecipitation assay buffer (RIPA, AbCam, Cambridge, MA) were added to 700 μL of the biologic prior to storing at −80°C. ACS and APS samples were diluted 50-fold prior to assaying due to high concentrations of IL-1Ra.

### Gene Expression

Cells were lysed 48 h after treatment and RNA was isolated using the RNeasy Mini kit (Qiagen, Germantown, MD). cDNA was prepared using the High Capacity cDNA Reverse Transcription kit (Applied Biosystems/ThermoFisher Scientific, Waltham, MA). Gene expression was determined for IL-1β, TNF-α, MMP-3, and MMP-13 using 18S as a reference gene. Equine primers and dual-labeled fluorescent probes [6-carboxyfluorescein (FAM) as the 5′ label (reporter dye) and ZEN and Iowa Black®FQ as the 3′ label (double quenching dyes)] for MMP-3, MMP-13, and 18S were designed using NCBI Primer-BLAST and Integrated DNA Technologies (IDT) PrimerQuest Tool (Table 1). Primers and probes for IL-1β (Ec04260298_s1) and TNF-α (Ec03467871_m1) were obtained from ThermoFisher Scientific's proprietary equine-specific gene expression assay database. Using 18S as a reference gene, relative expression of mRNA was determined using the ΔΔ Ct method. The efficiency of the primers and probe were determined by evaluating the slope of the log-linear phase and *r*^2^-values of the standard curve.

**Table 1:** Equine primer and probe sequences used for gene expression analyses.

**Gene**	**Primer and probe sequences**
18S, 18 small ribonucleic acid	Forward, 5′- GCCGCTAGAGGTGAAATTCT-3′ Reverse, 5′- TCGGAACTACGACGGTATCT−3′ Probe, 5′- AAGACGGACCAGAGCGAAAGCAT-3′
IL-6, interleukin 6	Forward, 5′- CTGCTCCTGGTGATGGCTAC-3′ Reverse, 5′- GCAGGTCTCCTGATTGAACC-3′ Probe, 5′-ACAGCAAGGAGGTACTGGCAGAAA-3′
MMP-3, matrix metalloproteinase 3	Forward, 5′-ATGGACCTTCTTCAGGACTACC-3′ Reverse, 5′-GACCGACATCAGGAACTCCG-3′ Probe, 5′-TGACACTGTGGAGGTGATGCACAA-3′
MMP-13, matrix metalloproteinase 13	Forward, 5′-ACAAGCAGTTCCAAAGGCTAC-3′ Reverse, 5′-CTCGAAGACTGGTGATGGCA-3′ Probe, 5′-AGACCCGAACCCTAAACATCCCAA-3′

*Primer and probe sequences for IL-1β and TNF-α are not available as they were obtained from ThermoFisher Scientific's proprietary equine-specific gene expression assay database*.

### Statistical Analysis

Histograms of data were visually inspected for a Gaussian distribution and a Shapiro–Wilk test was performed to determine normal distribution prior to statistical analysis. Data that were not normally distributed were analyzed using non-parametric methods. Comparisons between two groups (control and stimulated control) were made using a Wilcoxon rank sum test. Comparisons between all six groups were made using a mixed effects model with horse considered as a random effect and treatment considered as a fixed effect. Pairwise comparisons were made with Tukey's *post-hoc* test. Statistical analysis was performed using JMP14. The level of significance was set at *p* < 0.05.

## Results

### Cytokine Quantification in Autologous Blood Products

**Figure 1:**
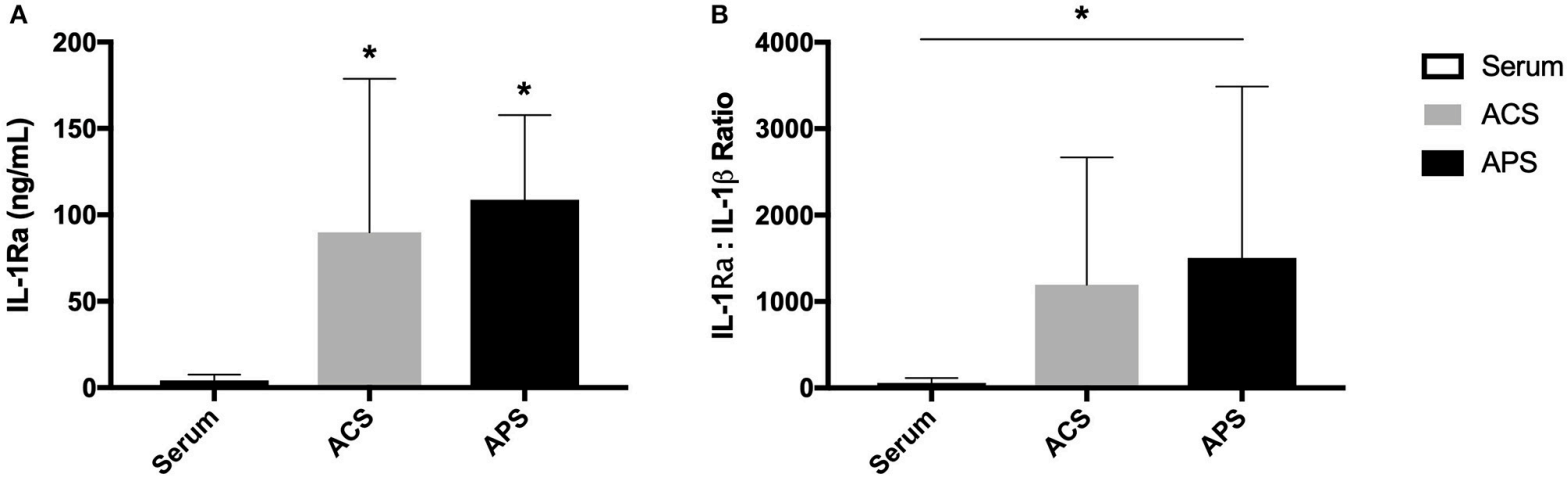
**(A)** Serum, ACS, and APS concentrations of IL-1Ra (ng/mL) and **(B)** the ratio of IL-1Ra to IL-1β in serum, ACS, and APS. Mean (±SD) for *n* = 6 horses shown. *Denotes significant differences between serum and ACS or APS, *p* < 0.05.

**Figure 2:**
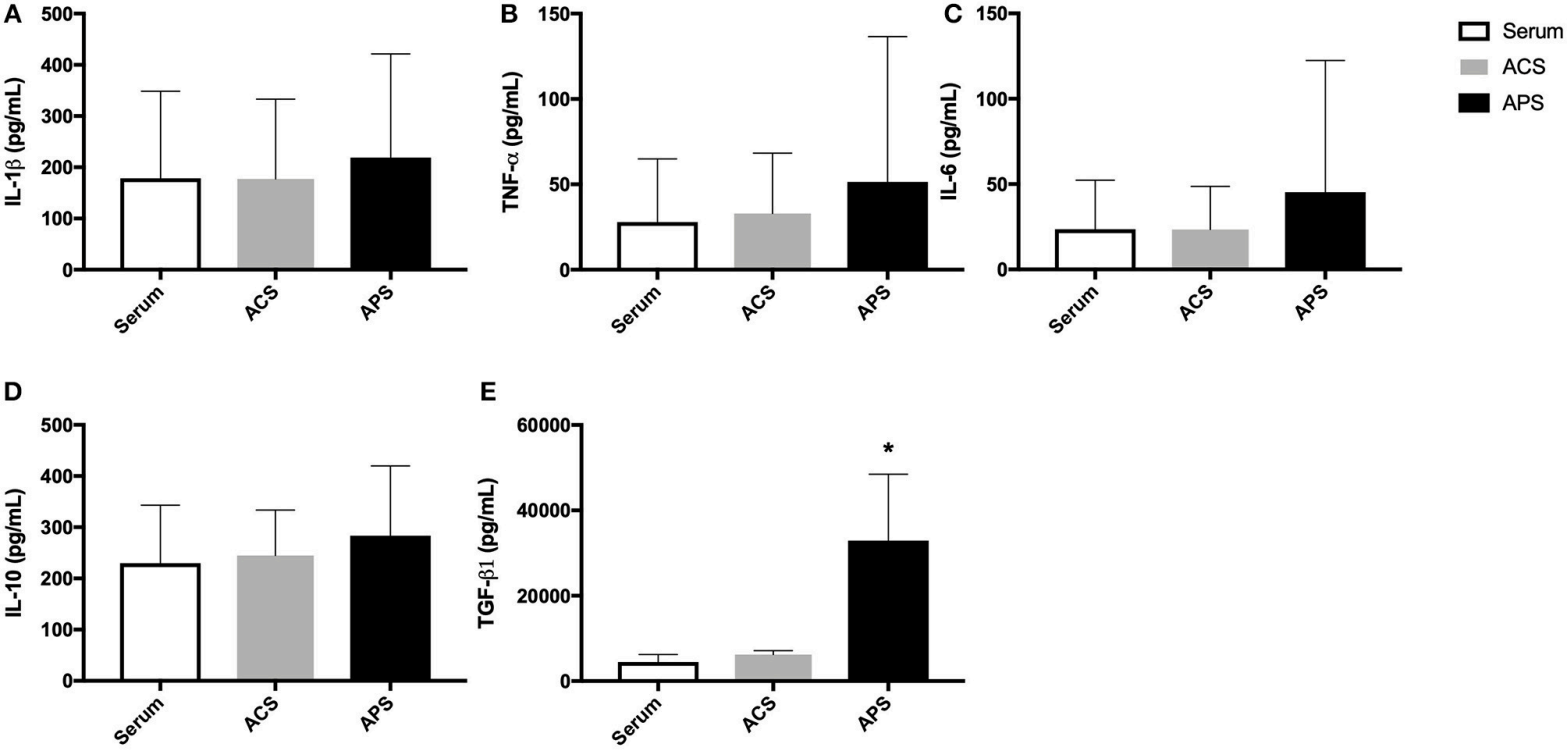
Serum, ACS, and APS concentrations of **(A)** IL-1β (pg/mL), **(B)** TNF-α (pg/mL), **(C)** IL-6 (pg/mL), **(D)** IL-10 (pg/mL), and **(E)** TGF-β1 (pg/mL). Mean (±SD) for *n* = 6 horses shown. *p < 0.05.

The mean (±SD) concentration of IL-1Ra was significantly increased in ACS (89.9 ± 88.8 ng/mL) and APS (108.8 ± 48.9 ng/mL) compared to serum (4.0 ± 3.4 ng/mL) (Figure 1). Additionally, the mean (±SD) IL-1Ra:IL-1β ratio was significantly higher in APS (1503.6 ± 1984.1 ng/mL) compared to control (55.0 ± 59.9 ng/mL) (Figure 1). The concentration of TGF-β was significantly increased in APS (32914.60 ± 15554.0 pg/mL) compared to serum (4486.35 ± 1763.5 pg/mL) and ACS (6230.20 ± 900.5 pg/mL). The concentration of other cytokines including IL-1β, IL-6, TNF-α, and IL-10 were similar in serum, ACS, and APS (Figure 2).

### Cytokine Quantification in Stimulated Chondrocyte Cultures

**Figure 3:**
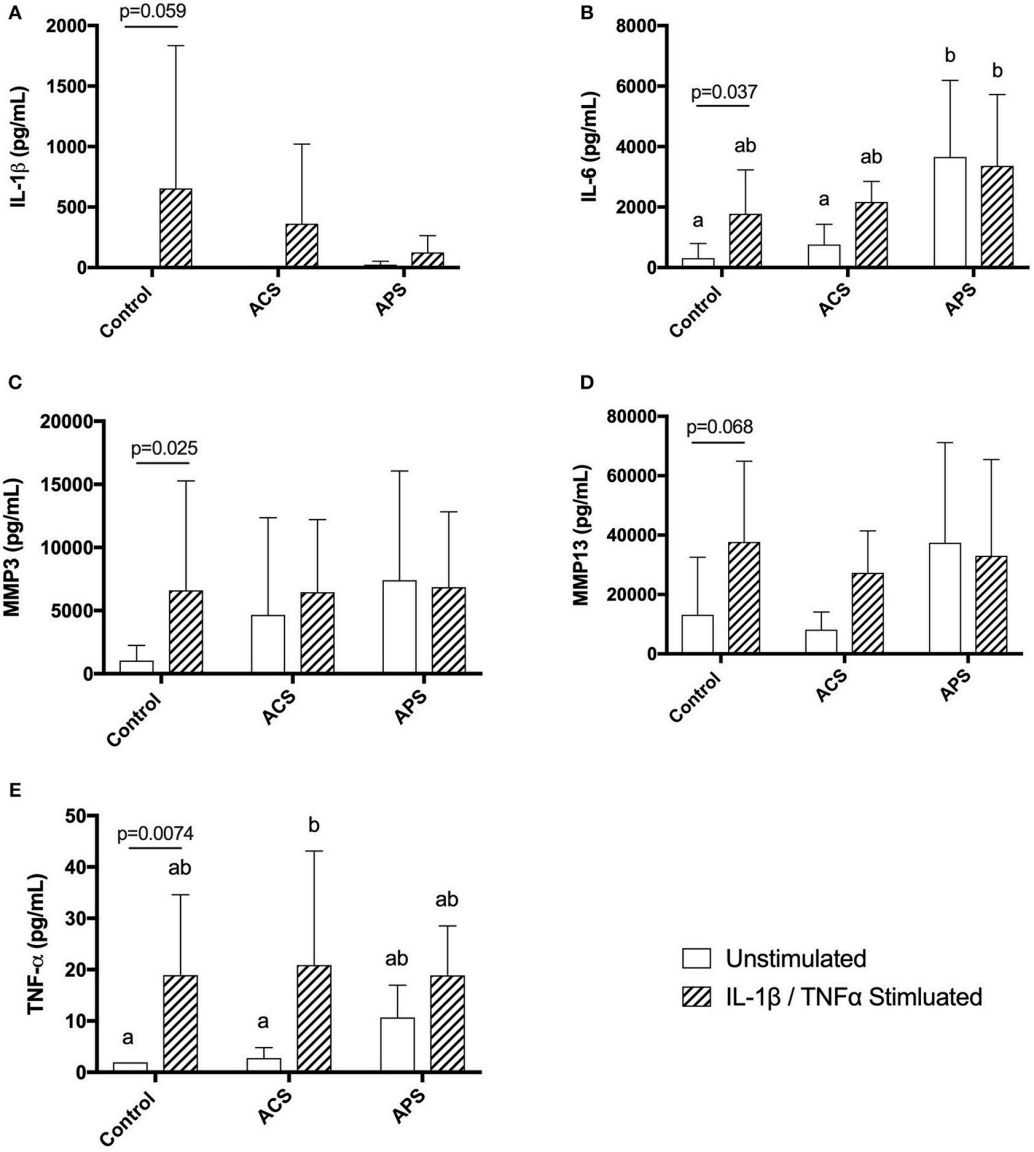
Supernatant concentrations of quantified cytokines **(A)** IL-1β, **(B)** IL-6, **(C)** MMP-3, **(D)** MMP13, and **(E)** TNF-α in control, ACS-treated, or APS-treated chondrocytes either with or without IL-1β/TNF-α stimulation after a 48 h culture period. Lines and *p*-values denote differences between unstimulated and stimulated chondrocytes only. Mean (±SD) for *n* = 6 horses shown. Different letters denote significant differences between all groups, *p* < 0.05.

Cytokine concentrations in supernatants from unstimulated control chondrocytes and IL-1β/TNF-α stimulated control chondrocytes were first compared to determine chondrocyte response to stimulation. Stimulation of control chondrocytes led to increased concentrations of IL-1β (*p* = 0.059), IL-6 (*p* = 0.037), TNF-α (*p* = 0.0074), MMP-3 (*p* = 0.025), and MMP-13 (*p* = 0.068) compared to unstimulated control chondrocytes (Figure 3).

When pretreatment with ACS or APS was considered, IL-1β concentration in culture supernatants was decreased in APS-treated chondrocytes compared to untreated controls, however, this did not reach statistical significance (Figure 3). IL-6 was significantly increased in unstimulated chondrocyte cultures pretreated with APS compared to unstimulated control chondrocytes and unstimulated chondrocytes pretreated with ACS. IL-6 was also significantly increased in stimulated chondrocyte cultures pretreated with APS compared to unstimulated control chondrocytes (Figure 3). MMP-3 and MMP-13 concentrations were increased in both unstimulated and stimulated chondrocyte cultures pretreated with ACS and APS compared to unstimulated control chondrocytes, however, these increases were not statistically significant (Figure 3). TNF-α concentration was significantly increased in stimulated chondrocyte cultures pretreated with ACS. TNF-α concentrations were also increased in unstimulated chondrocytes pretreated with ACS and unstimulated and stimulated chondrocytes pretreated with APS, however, none of these increases were statistically significant (Figure 3).

**Figure 4:**
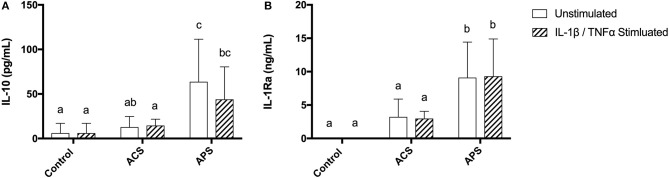
Supernatant concentrations of anti-inflammatory cytokines **(A)** IL-10 and **(B)** IL-1Ra in control, ACS-treated, or APS-treated chondrocytes with or without IL-1β/TNF-α stimulation after a 48 h culture period. Mean (±SD) for *n* = 6 horses shown. Different letters denote significant differences between groups, *p* < 0.05.

Similar to the APS product, the concentration of IL-1Ra was significantly increased in the supernatants of both unstimulated and stimulated chondrocytes pretreated with APS compared to control and ACS pretreated chondrocytes (Figure 4). IL-10 concentration in the supernatants of both unstimulated and stimulated chondrocytes pretreated with APS was also significantly increased compared to control and ACS pretreated chondrocytes (Figure 4).

### Gene Expression in Stimulated Chondrocytes

**Figure 5:**
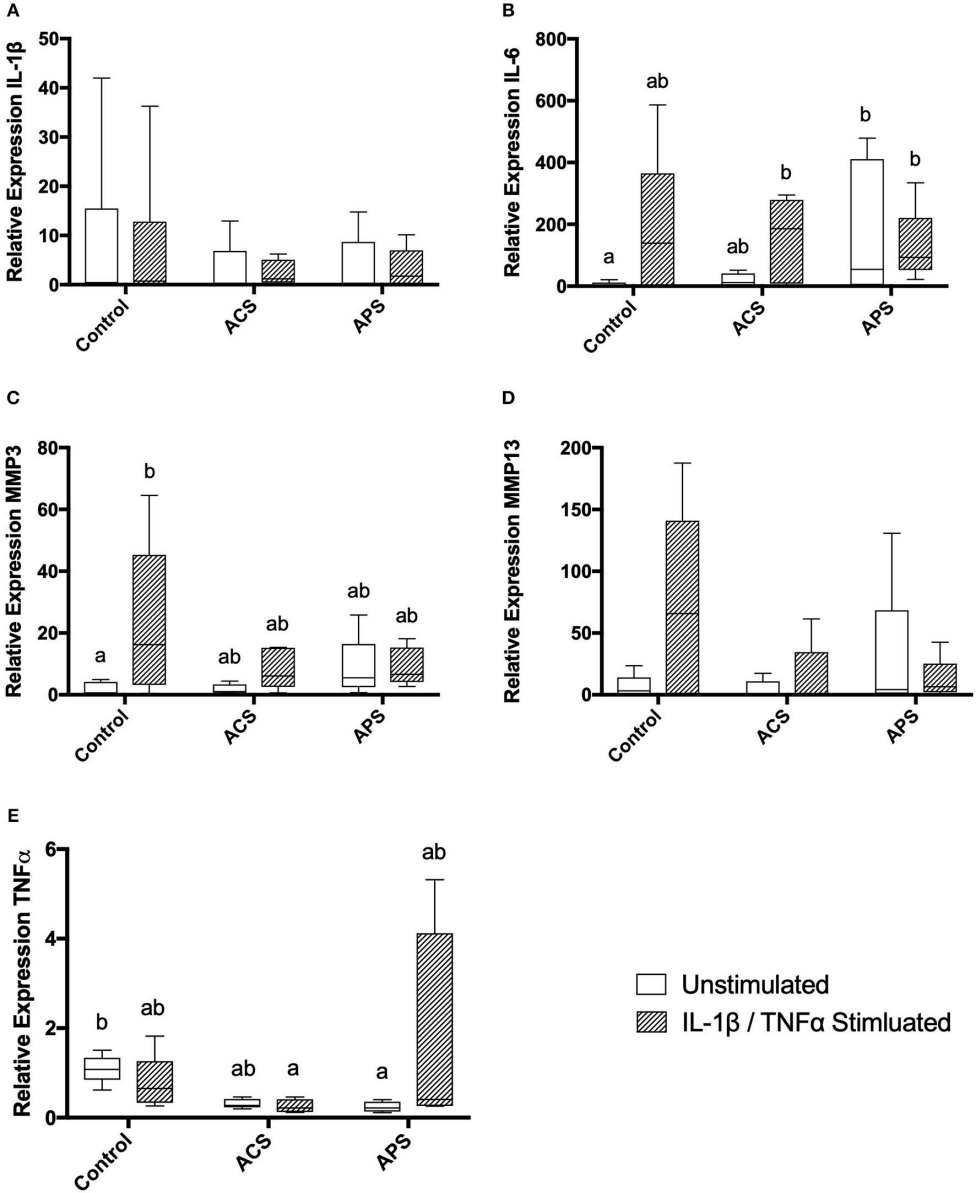
Relative mRNA expression of **(A)** IL-1β, **(B)** IL-6, **(C)** MMP-3, **(D)** MMP-13, and **(E)** TNF-α in control, ACS-treated, or APS-treated chondrocytes with or without IL 1β/TNF-α stimulation after a 48-h culture period. Different letters denote significant differences between groups, *p* < 0.05.

Gene expression was assessed in chondrocytes after 48 h of treatment. No significant changes were seen in expression of IL-1β amongst the treatment groups. Similar to changes in supernatant concentrations of IL-6, expression of IL-6 was increased in stimulated chondrocytes, unstimulated, and stimulated chondrocytes pretreated with ACS and unstimulated and stimulated chondrocytes pretreated with APS compared to unstimulated control chondrocytes (Figure 5). Expression of MMP-3 was significantly increased in stimulated control chondrocytes only, with no significant differences noted in expression of MMP-13 (Figure 5). Expression of TNF-α was significantly lower in unstimulated chondrocytes pretreated with APS and in stimulated chondrocytes pretreated with ACS when compared to unstimulated, control chondrocytes (Figure 5).

## Discussion

Autologous protein solution, an orthobiologic that concentrates platelets and anti-inflammatory cytokines, has been shown to improve clinical symptoms associated with knee osteoarthritis in human patients (12, 13). Similarly, in veterinary patients, single intra-articular APS injections have been shown to improve clinical signs in dogs and horses with osteoarthritis (14, 15). Although APS is thought to mitigate the inflammatory cascade within joints, the mechanism by which this occurs has not been fully elucidated. In this study we sought to quantify the concentration of several cytokines involved in the inflammatory cascade of chondrocytes in equine APS and evaluate the effect of APS on stimulated equine chondrocytes. We found that APS had significantly increased IL-1Ra compared to serum (100-fold increase) and importantly had a significantly increased IL-1Ra:IL-1β ratio (1,500:1). Interleukin-1 receptor antagonist protein (IL-1ra or IRAP), is a potent anti-inflammatory protein that binds type IL-1 receptors and competitively inhibits IL-1α and IL-1β, both of which initiate a pro-inflammatory cascade in osteoarthritis through activation of NF-κB (16). Previous studies have shown that 10- to 100-fold excess amounts of IL-1Ra are needed to effectively inhibit IL-1 signaling (17). King et al. also found that higher IL-1Ra:IL-1β were significantly correlated with improved Western Ontario and McMaster Universities Osteoarthritis Index (WOMAC) pain scores in humans with knee osteoarthritis following injection with APS (18).

In this study, the concentration of the anti-inflammatory cytokine IL-10 was not significantly different in serum, ACS, or APS. These results differ from previously published results in which equine ACS and APS were shown to have significantly increased quantities of IL-10 compared to serum (15, 19). Bertone et al. found that APS had an average of 3,000 ng/mL of IL-10 compared to an average of 970 ng/mL of IL-10 in serum, resulting in a 3.4-fold difference, whereas we found that serum, ACS, and APS all contained ~250 pg/mL of IL-10 (15). Hraha et al. also found that equine ACS had significantly more IL-10 than serum, however, the overall IL-10 concentration in ACS was ~250 pg/mL, significantly lower than Bertone et al. described in equine APS (15, 19). The major differences in IL-10 quantification may be due to the use of different assays. In this study we used a fluorescent bead-based multiplex assay validated for use in equine samples while both previous reports describe the use of a commercial IL-10 ELISA. In human APS, a significant increase in IL-10 over baseline has been noted, however, APS only contained ~5.2 pg/mL of IL-10 compared to 1.1 pg/mL of IL-10 in serum (20).

We found that APS contained significantly more TGF-β1 than serum or ACS. The fold increase in the concentration of TGF-β1 in our APS (~7X) is reflective of the fold increase in TGF-β1 found in human APS, although the mean baseline concentration of human TGF-β1 is considerably higher (25,717 pg/mL) (20). The concentration of TGF-β1 in ACS in our study was similar to that reported by Nielsen et al. but higher than that reported by Hraha et al. which once again may be reflective of inter-animal variability and differences in assays (19, 21). TGF-β1 is an important growth factor involved in the development, growth and maintenance of articular cartilage, although its role in osteoarthritis has not been completely elucidated (22). Several studies have shown that TGF-β1 supports chondrogenic differentiation of stem cells (23, 24) and upregulates hyaluronan synthesis in cultured equine chondrocytes (25). However, TGF-β1 has also been implicated in osteophyte formation and synovial fibrosis, mainly in murine studies (26, 27). The effect of increased intra-articular concentration of TGF-β1in the horse remains unknown and further studies are indicated.

Since the therapeutic value of IL-1Ra is purported to rely on high IL-1Ra to IL-1β ratio, it is ideal that APS preparation does not lead to a concurrent increase in IL-1β. In this study, we did not find any significant increases in the concentration of the pro-inflammatory cytokines IL-1β or TNF-α in ACS or APS compared to serum. Similar results have been reported in human APS (20). However, Hraha et al. described increased concentrations of IL-1β or TNF-α in preparations of equine ACS compared to serum (19) and Rutgers et al. found similar results in human ACS (28).

In addition to APS having increased concentrations of IL-1Ra, the supernatants collected from chondrocyte cultures pretreated with APS had significantly increased concentrations of IL-1Ra following the 48-h culture period. The concentration of IL-10 in supernatants collected from APS-treated cultures was also significantly increased. It is possible that IL-1Ra and IL-10 persisted in APS-treated cultures longer than ACS-treated cultures as the concentrations of IL-1Ra and IL-10 in the APS and ACS preparations were similar prior to adding them to cultures. Alternatively, it is possible that cytokines in APS induce chondrocytes to produce IL-1Ra and/or IL-10. Previously, Jenkins and Arend found that IL-1Ra production was induced by IL-4 (29). Currently, the mechanism leading to increased chondrocyte synthesis of IL-10 is unknown (30), however, it is certainly feasible that IL-10 is being induced by components of APS.

Interleukin-6 gene expression and secretion was significantly increased in both unstimulated and stimulated chondrocytes pretreated with APS. The role of IL-6 in joint disease remains uncertain. Interleukin-6 has been found to be increased in osteoarthritic joints (31, 32) but despite initial studies suggesting that IL-6 was simply a catabolic cytokine (33) several recent studies have suggested a more modulatory role in equine cartilage (34, 35). Recently, studies evaluating the role of IL-6 in equine chondrocytes have shown IL-6 to be pro-chondrogenic and not associated with a catabolic cascade (34, 35). It is possible that elevated levels of IL-6 in APS-treated chondrocytes would have a beneficial role in inflamed and/or osteoarthritic joints, however, further investigations into the broad effects of IL-6 are warranted.

Autologous protein solution treatment of stimulated human chondrocytes was shown to significantly decrease MMP-13 production by Woodell-May et al. (20). In this study, supernatant MMP-3 or MMP-13 concentrations were not significantly affected by APS treatment. Gene expression of MMP-13 was decreased in stimulated APS-treated chondrocytes compared to stimulated control chondrocytes, however, this did not reach statistical significance. Lack of statistical significance in gene expression may be explained, in part, due to the large variability amongst the horses. Interestingly, MMP3 and MMP13 concentrations were increased in the supernatants of unstimulated APS-treated chondrocytes, although these increases were not statistically significant. It is possible that APS contains MMP3 and MMP13 that is produced during processing, however, this was not specifically tested.

Study limitations include the use of a monolayer chondrocyte culture. The joint is a complex organ with cartilage, synovium, and subchondral bone all playing a role in the inflammatory cascade. Further studies evaluating the effect of APS using cartilage explant and synovium co-cultures are warranted. Further limitations include the inability to quantify all cytokines and growth factors that may be playing a role in joint inflammation and degeneration. For example, we did not quantify the effect of APS on the ADAMTSs, a group of aggrecanases with well-known degradative effects on the ECM (36). Additionally, we did not quantify several growth factors that are concentrated in PRP such as PDGF and transforming growth factor-β (TGF-β). These growth factors have been shown to be concentrated in human APS and further investigations of equine APS should aim to quantify such factors (20, 37).

In conclusion, APS, a patient-side, combinatorial orthobiologic, has a significantly increased concentration of IL-1Ra without a concurrent increase in IL-1β concentration. After 48 h of culture, media from chondrocytes treated with APS contains significantly increased concentrations of IL-1Ra and IL-10, both of which are chondroprotective. Additionally, APS-treated cultures have increased concentrations of IL-6, a modulatory cytokine that may support chondrocyte homeostasis. However, our hypothesis that APS would be superior to ACS cannot be supported as APS treatment of chondrocytes did not decrease expression or synthesis of many pro-inflammatory cytokines in this *in vitro* model. Overall, APS effectively concentrates IL-1Ra without an incubation period and may be beneficial in the treatment of inflammatory conditions such as PTOA.

## Author Contributions

RL and WK contributed to study design, acquisition, analysis and interpretation of data, and preparation of the final manuscript. MD and KM contributed to acquisition of data. KO contributed to study design, acquisition, analysis and interpretation of data, and preparation of the manuscript. All authors approved the final manuscript.

### Conflict of Interest Statement

WK is an employee of Owl Manor Veterinary Inc. The remaining authors declare that the research was conducted in the absence of any commercial or financial relationships that could be construed as a potential conflict of interest.
